# A protocol for a feasibility randomised controlled trial to assess the difference between functional bracing and plaster cast for the treatment of ankle fractures

**DOI:** 10.1186/s40814-017-0125-z

**Published:** 2017-03-01

**Authors:** Rebecca S. Kearney, Nick Parsons, Dipesh Mistry, Jonathan Young, Jaclyn Brown, Joanne O’Beirne-Elliman, Matthew Costa

**Affiliations:** 1 Clinical Trials Unit, University of Warwick, CV4 7AL Gibbet Hill Road, Coventry, England; 2 Warwick Medical School, University of Warwick, CV4 7AL Gibbet Hill Road, Coventry, England; 3 University Hospitals Coventry and Warwickshire NHS Trust, CV2 2DX Clifford Bridge Rd, Coventry, England; 4 Trauma Unit, Kadoorie Centre, Level 3, John Radcliffe Hospital, OX3 9DU Oxford, UK

**Keywords:** Ankle fracture, Rehabilitation, Orthopaedic procedures

## Abstract

**Background:**

UK Hospital Episode Statistics 2013–2014 recorded 57,286 fractures of the lower limb including the ankle. This figure is expected to continue to increase due to a greater population of older adults. Following an ankle fracture, patients usually have their ankle immobilised with a plaster cast. This provides maximum support for the healing ankle but is associated with stiffness and muscle wasting. A Cochrane Review has concluded that functional bracing may reduce muscle wasting and speed recovery of ankle movement.

The aim of this study is to determine the feasibility of conducting a full randomised controlled trial in adults with an ankle fracture followed by functional bracing and exercises versus standard plaster cast care.

**Methods:**

This is a single-centre feasibility randomised controlled trial. All patients with a fractured ankle are potentially eligible. The trial will employ 1:1 random allocation, stratified by age and non-operative/operative management. Baseline demographic and pre-injury functional data, the Manchester-Oxford Foot and Ankle Questionnaire (MOXFQ) and Olerud and Molander Ankle Score (OMAS) will be collected alongside the EuroQol EQ-5D-5 L health-related quality of life questionnaire. A research associate will perform a clinical assessment and obtain X-rays in 6 weeks and 6 months post randomisation to record complications. Functional outcome and health-related quality of life will be collected in 6 weeks, 3 and 6 months post randomisation.

**Discussion:**

This feasibility trial will provide authoritative high-quality evidence to inform the design of a definitive trial in this important area.

**Trial registration, sponsorship and funding:**

This study is registered with the ISRCTN (ISRCTN17809322), assigned 5 November 2015 and approved by the NRES Committee (The Black Country, 15/WM/0340), protocol version 2.0 (17 November 2015). It is co-sponsored by the University Hospitals Coventry and Warwickshire NHS Trust and University of Warwick and funded by the NIHR Research for Patient Benefit (PB-PG-0614-34009). The trial sponsors have no direct involvement in any aspects of study design, conduct or decision to submit the report for publication.

**Electronic supplementary material:**

The online version of this article (doi:10.1186/s40814-017-0125-z) contains supplementary material, which is available to authorized users.

## Background

UK Hospital Episode Statistics 2013–2014 recorded 57,286 fractures of the lower limb including the ankle, affecting between 107 and 187 per 100,000 persons [1, 2]. The short-term impact of this injury results in physical impairments, including pain, stiffness, weakness and swelling. The longer-term impact often includes prolonged time off work, development of post-traumatic arthritis and psychological consequences of depression and anxiety [3].

The main bones of the ankle are the talus (in the foot) and the tibia and fibula (in the leg). Distally, the tibia and fibula are bound by a fibrous band called the syndesmosis and have ligament attachments on the inside of the ankle (deltoid ligament) and outside of the ankle (lateral collateral ligaments) which are important for ankle stability. Ankle fractures that occur below the level of the syndesmosis are usually managed with functional braces. Ankle fractures that occur at the level of the syndesmosis can lead to instability, with some requiring open reduction and internal fixation (ORIF) with the aim of restoring stability [3].

For ankle fractures that occur at the level of the syndesmosis, regardless of the decision to operate or not, the immediate rehabilitation has traditionally been plaster cast immobilisation and non-weight-bearing for several weeks. A cast provides maximum support; however, there are potential problems including the immediate impact on mobility for a period of around 6 weeks and risks associated with prolonged immobilisation such as muscle atrophy, deep vein thrombosis and joint stiffness. There are also long-term consequences, which can include prolonged gait abnormalities, persistent calf muscle weakness and an inability to return to previous activity levels. Alternative functional bracing may potentially address these issues [4]. However, it does not provide the same degree of support to the healing bones [3].

This topic was addressed by a Cochrane Review in 2012 [5]. It concluded that functional bracing may improve functional outcome, pain and improve ankle movement. However, these potential advantages needed to be balanced against the increased incidence of adverse events. Consequently, future research was recommended to confirm the clinical and cost-effectiveness of functional bracing following an ankle fracture [5]. In light of the large personal and societal cost associated with the injury, this gap in the evidence is a clear priority.

In 2010, an orthopaedic trauma network (AOUK) undertook a research priority exercise [6]. One of the top priority questions was to establish if there is a clinical advantage to wound healing and ankle function of different rehabilitation plans following an ankle fracture.

The aim of this feasibility trial is to derive sufficiently precise estimates of unknown study population parameters (e.g. outcome variability) such that the feasibility of conducting a full randomised controlled trial (RCT) can be determined. The full RCT will assess the difference in the Manchester-Oxford Foot and Ankle Questionnaire (MOXFQ) in 6 months after injury between adults with an ankle fracture followed by functional bracing and exercises versus standard plaster cast care.

## Methods

### Research question

Is it feasible to conduct a full RCT to assess the difference in the MOXFQ in 6 months after injury between adults with an ankle fracture followed by functional bracing and exercises versus standard plaster cast care?

### Objectives

1. Evaluate the distributional properties of the MOXFQ in order to estimate the likely sample size required for a full RCT
2. Evaluate the number of eligible patients within the recruiting site
3. Evaluate the willingness of clinicians to recruit participants (the proportion of eligible patients who are offered participation in the study)
4. Evaluate the willingness of participants to be randomised (the proportion of eligible patients who agree to participate in the study)
5. Evaluate the follow up and response rates to questionnaires
6. Refine the statistical analysis plan to provide the most efficient and sensitive analysis
7. To discuss the feasibility trial at a national consensus meeting to inform the design of a full RCT

### Design

This is a single-centre feasibility randomised controlled trial. All patients with a fractured ankle under the care of an orthopaedic consultant in a single UK major trauma centre are potentially eligible. The trial will employ 1:1 random allocation, stratified by age and operative/non-operative management, implemented using a remote, independent telephone randomisation service. A total of 50 participants will be randomised.

All participants will follow a standardised protocol for both treatments. Baseline demographic data and pre-injury functional data using the MOXFQ and Olerud and Molander Ankle Score (OMAS) will be collected alongside the EuroQol EQ-5D-5 L health-related quality of life questionnaire.

A research associate will perform a clinical assessment and obtain X-rays in 6 weeks and 6 months to record any early and late complications. Functional outcome and health-related quality of life will be collected in 6 weeks, 3 and 6 months post randomisation.

### Participants

In standard clinical practise, a patient will first present to the health professional with a history of ‘twisting’ on the ankle or ‘falling’ [2]. The patient will then have an initial X-ray, followed by a discussion with the health professional about their injury that requires surgery and the type of immobilisation required.

In this clinical trial, all adult patients with an ankle fracture for which the treating clinician would traditionally treat the patient in a plaster cast will be considered. In this pragmatic design, this is the primary and most important inclusion criteria. Patients who lack capacity under the Mental Capacity Act 2005, have open ankle fractures, who require close contact casting, have pathological fractures (e.g. known metastatic disease) or any other concurrent lower limb injury that would affect the functional outcome measures (including bilateral ankle fractures and syndesmosis injury requiring surgery) will be excluded.

Screening logs will be collected to assess the main reasons for patient exclusions, as well as the number of patients unwilling to take part. These broad eligibility criteria will ensure that the results of the study can readily be generalised to the wider patient population.

## Intervention and comparator

### All patients

All patients having ankle fixation surgery will have the preferred technique of the operating surgeon. A copy of the ‘operating record’ will form part of the trial dataset, including the grade and experience of the surgeon. All patients will then be placed in a back slab until the stitches are removed 10 days post-operatively, at which point the intervention will be applied.

All patients not receiving surgery will be approached to take part in the trial on first presentation to the trauma team fracture clinic.

### Control group: standard plaster cast

All patients in the control arm will be immobilised with a plaster cast, as per standard practise; the cast may be Plaster of Paris or fibreglass. To monitor compliance, all patients will be asked to complete a daily dairy of how much they bear weight.

### Intervention group: functional bracing

All patients in the intervention arm will be fitted with a removable functional brace. A functional brace is a plastic shell in the shape of a boot, covering the whole foot and leg below the knee, and it has an inner foam liner that is held in place with Velcro. The exact choice of removable functional brace (manufacturer, model, etc.) will reflect the usual hospital stock available, reflecting the pragmatic nature of this study.

Whilst wearing the functional brace, patients will be encouraged to remove their functional brace to complete active unloaded ankle range of movement exercises three times per day, completing ten repetitions on each occasion. To monitor compliance and intervention fidelity, all patients will be asked to complete a daily exercise diary.

Six weeks post treatment, all patients (operative and non-operative) will receive the same standardised, written physiotherapy advice detailing the exercises they need to perform. In this pragmatic trial, any other rehabilitation input beyond the written physiotherapy advice (including a formal referral to physiotherapy) will be left to the discretion of the treating clinicians. However, a record of any additional rehabilitation input (type of input and number of additional appointments) together with a record of any other investigations/interventions will be requested as part of the 3- and 6-month follow-up data.

### Post randomisation withdrawals

Participants may be discontinued from the trial treatment and/or the trial at any time without prejudice. Unless a participant explicitly withdraws their consent, they will be followed up wherever possible and data will be collected as per the protocol until the end of the trial.

### Concomitant illness

Details of any concomitant illness will be recorded at trial entry and all except those outlined in the exclusion criteria are permitted during the trial.

## Outcomes

### Patient-centred outcomes

Baseline data will be collected by the research associates from all participants and will include age, sex, general medical history and pre-injury functional and health status. This will be followed by ascertaining the current function and health status of the patient.

Link to objectives 1 and 6: evaluate the distributional properties of the MOXFQ in order to estimate the likely sample size required for a full RCT. Refine the statistical analysis plan to provide the most efficient and sensitive analysis.

The primary functional status outcome measure for this study is the MOXFQ. Other commonly used foot and ankle scores include the Olerud Molander Ankle Score (OMAS) and American Orthopaedic Foot and Ankle Score (AOFAS); however, they lack a methodologically robust approach in their development, in contrast to the MOXFQ. The MOXFQ is a validated questionnaire which is self-reported (filled in by the patient). It contains 16 items, each with five response options comprising three separate underlying dimensions: walking/standing problems (seven items), foot pain (five items) and issues related to social interaction (four items). Item responses are each scored from 0 to 4, with 4 representing the most severe state. The scale scores representing each dimension are produced by summing the responses to each item within that dimension. Raw scale scores are then converted to a metric (0–100; 100 = most severe) [7]. This will be collected to allow the distributional properties to be evaluated for a later sample size calculation.

Link to objective 2: evaluate the follow up and response rates to questionnaires

The secondary objective measures proposed for the full RCT are health-related quality of life outcome measure for this study is the EQ-5D-5 L. The EQ-5D-5 L is a validated, generic health-related quality of life measure consisting of five dimensions each with a five-level answer possibility. Each combination of answers can be converted into a health utility score. It has good test-retest reliability, is simple for patients to use and gives a single preference-based index value for health status that can be used for broader cost-effectiveness comparative purposes [8]. Although cost-effectiveness analysis is not being carried out as part of this study, it is planned to be included in the definitive study. The methods will adhere to the recommendations of the NICE reference case, which included collection of EQ-5D-5 L. A record of all complications will also be included. The team will evaluate the proportion of these questionnaires that are completed and returned.

Radiographic evaluation of routine pre- and post-injury X-rays (taken at baseline, 6 weeks and 6 months after the injury) will also be collected from the trial team. Standard measurements of joint congruence, fracture angulation, fibular shortening and subluxation will be assessed [9]. We will again evaluate the proportion of these returned.

Whilst the exact number of appointments varies, most patients will be kept under review for 6 months as part of normal practise. The trial procedures will therefore mirror usual care with collection of all outcome measures at baseline, 6 weeks and 6 months face to face by a research associate in the follow-up fracture clinic. This will be alongside postal follow up in 3 months post injury. We will use techniques common in long-term cohort studies to ensure minimum loss to follow-up, such as collection of multiple contact addresses and telephone numbers, mobile telephone numbers and email addresses [10]. Refer to Table 1 for participant timeline.

**Table 1:** Schedule of enrolment, interventions and assessments

	Enrolment	Allocation	Post-allocation		
Timepoint			6 weeks (±2 weeks)	3 months (±1 month)	6 months (±1 month)
Enrolment					
Eligibility screen	X				
Informed consent	X				
Allocation		X			
Interventions					
Functional brace		X	X		
Plaster cast		X	X		
Assessments					
Demographic data	X				
EQ5D-5 L/MOXFQ/OMAS	X		X	X	X
Complications			X	X	X
Exercise diary			X		
Physiotherapy input			X	X	X
Radiographs		X	X		X

### Feasibility outcomes

Link to objectives 3, 4 and 5: evaluate the number of eligible patients within the recruiting site; evaluate the willingness of clinicians to recruit participants (the proportion of eligible patients who are offered participation in the study); evaluate the willingness of participants to be randomised (the proportion of eligible patients who agree to participate in the study).

As part of the trial processes, designated research associates will attend daily trauma meetings and fracture clinics held at the trial site. At these locations, they will identify potentially eligible patients who will be entered on a screening log. These logs will contain details of every patient who attends the trial site with an ankle fracture. The TMG will then monitor the number of screened patients who were eligible and offered participation for the trial and the number offered the trial who agree to participate.

Link to objective 7: to discuss the feasibility trial at a national consensus meeting to inform the design of a full RCT.

All the above outcomes will be discussed at a national consensus meeting to discuss more fully with patient, clinician and research representatives the feasibility of conducting a full RCT.

## Sample size

As this is a feasibility study, a formal power analysis for the sample size will not be undertaken, as the study is not designed to test a specific null hypothesis and infer significance to any observed treatment differences. Instead, we adopt a more informal approach, based on the aims of (i) assessing the distributional properties of the MOXFQ (specifically the variance) and (ii) assessing the likely participation rate (the proportion of eligible patients who are likely to agree to participate in the study). For the first aim, 50 participants in total, 25 in each arm, will be analysed. A sample size of 50 will allow us to estimate a participation rate of 75% to within a 95% confidence interval of ±12%. The data obtained from this study will inform the power analysis for the full study [11, 12].

## Recruitment

Potentially eligible participants will be identified by the patients clinical care team in the emergency department, fracture clinics and trauma wards. The trauma team will undertake the initial approach, explaining that a study of ankle fracture rehabilitation is being conducted. If the patient is willing to be approached, a suitably qualified person will then provide verbal and written information about the study.

This feasibility study will specifically inform the recruitment rate for the main trial. However, recruitment has been estimated on audit data at the lead centre [13] and previous research in a related area completed at Warwick Clinical Trials Unit [14]. In the lead centre audit, 176 ankle fractures presented for an ankle pathway over a 16-month period, approximately 11 per month.

## Assignment of interventions

Pre-randomisation eligibility checks will be carried out to ensure that patients meet the eligibility criteria. Written informed consent for entry into the trial will be obtained prior to randomisation.

Allocation will be made using a secure, centralised computer-generated allocation sequence, using a telephone-based, independent randomisation service. A suitably qualified member of the research team will inform the treating clinical team of the allocated treatment.

Stratification on the basis of age will be used to discriminate between younger patients with normal bone quality sustaining high-energy fractures (under 50 years), and older patients (over 50 years) with low-energy (fragility) fractures related to osteoporosis [15, 16]. A second stratification, based on whether the patient receives surgery or not, will also be used. This will ensure that any effect related to severity of injury is equally distributed amongst the trial arms.

## Blinding

As the type of rehabilitation used is clearly visible, the participants cannot be blinded to their treatment. In addition, the treating surgeons cannot be blinded to the treatment but will take no part in the assessment of patients.

The questionnaire data will be collected and entered onto the trial central database via postal mechanisms by a research assistant/data clerk in the trial central office. All X-ray data will be reviewed by two independent assessors. The independent assessors will have no part in study procedures and will therefore be blinded to treatment allocation.

## Data

### Collection, management and monitoring

All electronic identifiable information will be held on a secure, password-protected database accessible only to essential personnel. Paper forms with identifiable information will be held in secure, locked filing cabinets within a restricted area. Personal data collected during the trial will be handled and stored in accordance with the 1998 Data Protection Act. Participants will be identified by a code number only. Direct access to source data/documents will be required for trial-related monitoring by authorised personnel only. All paper and electronic data will be retained for at least 10 years after completion of the trial.

The trial coordinator and data clerk will check and enter the data onto the trial database, which will be developed by the Programming Team at Warwick Clinical Trials Unit. Promotion of data quality will be achieved through implementation of a data management plan held at the Warwick Clinical Trials Unit.

### Analysis

Members of the trial management group, University of Warwick, University of Oxford and University Hospitals Coventry and Warwickshire NHS Trust, will have access to the final trial dataset. Given the small sample size, it is unlikely that formal hypothesis testing will be useful or informative. This data will be analysed using the software package STATA and will be presented using standard methods for statistical summaries of discrete and continuous data sets.

All data will be analysed and reported in accordance with the CONSORT statement. Baseline data will be summarised to check comparability between treatment arms and to highlight any characteristic differences between those individuals in the study, those ineligible and those eligible but withholding consent. As this is a feasibility study, the analysis will be performed through descriptive statistics rather than formal hypothesis testing. Results will be pooled and summarised using standard methods as appropriate and presented graphically to aid interpretation. Standard statistical summaries and graphical plots showing correlations will be presented for the primary outcome measure (MOXFQ) and all secondary outcome measures.

An exploratory linear regression analysis will also be undertaken in order to explore treatment group effects, after adjusting for the effects of patient age, fixation/non-operative management and gender.

Temporal patterns of any complications will be presented graphically, and if appropriate, a time-to-event analysis (Kaplan-Meier survival analysis) will be used to assess the overall risk and risk within individual classes of important complications (e.g. infection). The decision about whether to undertake a time-to-event analysis will be data-dependent, that is it will be dependent on the number of complications reported by study participants.

It seems likely that some data may not be available due to voluntary withdrawal of participants, lack of completion of individual data items or general loss to follow-up. Where possible, the reasons for data ‘missingness’ will be ascertained and reported. Although missing data is not expected to be a problem for this study, the nature and pattern of the missingness will be carefully considered—including in particular whether data can be treated as missing completely at random or missing at random. Reasons for ineligibility, non-compliance, withdrawal or other protocol violations will be stated and any patterns summarised. More formal analysis, for example using logistic regression with ‘protocol violation’ as a response is unlikely to be useful given the small sample size but may be considered if appropriate to aid interpretation.

Statistical methods for calculating the definitive RCT sample size will follow the conventional approach using four parameters: type I error, power, assumptions in the control group (response rate and standard deviation) and expected treatment effect.

## Monitoring

As this is a single-site feasibility study, a data monitoring committee and trial steering committee have not been convened. There is no planned interim analysis.

The trial will be monitored by the trial management group, who are employed by the sponsor and supported by the funder. The trial management group will meet monthly to discuss progress against key milestones and be responsible for reporting to the sponsor and funder.

### Reporting of SAE

An adverse event (AE) is defined as any untoward medical occurrence in a participant which does not necessarily have a causal relationship with this treatment/intervention. All AEs will be listed on the appropriate case report form (CRF) for routine return to the ‘AIR’ central office.

A serious adverse event is an AE that fulfils one or more of the following criteria:

- Results in death
- Is immediately life-threatening
- Requires hospitalisation or prolongation of existing hospitalisation
- Results in persistent or significant disability or incapacity
- Is a congenital abnormality or birth defect
- Is an important medical condition

All serious adverse events (SAE) will be entered onto the serious adverse event reporting form and faxed to the ‘AIR’ central office within 24 h of the investigator becoming aware of them. Once received, causality and expectedness will be confirmed by the chief investigator. SAEs that are deemed to be unexpected and possibly related to the trial interventions will be notified to the Research Ethics Committee (REC) within 15 days. All such events will be reported to the trial management group at their next meeting.

SAEs that may be expected as part of the interventions and that do not need to be reported to the coordinating centre are complications of anaesthesia or surgery (e.g. wound complications, infections, damage to a nerve or blood vessel and thromboembolic events) and secondary operations for or to prevent infection, malunion, non-union or for symptoms related to the metalwork. These will be recorded on the participant’s CRF. All participants experiencing SAEs will be followed-up as per protocol until the end of the trial.

### Auditing

We will institute a rigorous programme of quality control. The chief investigator in conjunction with the trial coordinator will be responsible for ensuring adherence to the trial protocols at the trial sites. Quality assurance checks will be undertaken by University of Warwick to ensure integrity of randomisation, study entry procedures and data collection. The University has a quality assurance manager who will monitor this trial by conducting regular (yearly or more if deemed necessary) inspections of the Trial Master File.

## Ethics and dissemination

This study was approved by the NRES Committee – The Black Country, REC ref: 15/WM/0340 Version 1.0 approved 21 October 2015, and Protocol 2.0 was approved 17 November 2015. All changes are documented in the trial site file and communicated with the REC, lead site, sponsor and funder.

The dissemination strategy will consist of three strands. The first will ensure that patients and public are informed of the trial results via publication and online resources; the second will engage practitioners and health care planners nationally via publication and conference presentations and the third will involve consulting with networks for future planning of a full application.

## Discussion

This feasibility study will answer the question regarding whether a definitive UK multi-centre RCT in this important area can be achieved. It will also provide authoritative high-quality evidence to inform the definitive trial design.

Randomised trials in a trauma setting are often laden with barriers and uncertainties. Specifically, for this trial, it is imperative that processes are refined to identify patients as they present into a busy clinical environment and once identified are provided with the trial information. Willingness of clinicians to include identified participants will be a key driver determining the future feasibility of this study alongside the willingness of patients to participate. These aspects will be carefully monitored by the trial management group on a monthly basis, and key documents will be refined in response to feedback from the lead site.

Understanding and reducing potential barriers to enrolment from both a clinician and patient perspectives is an important factor alongside the quantitative measures of the proportion of eligible patients who consent/clinicians put forward for enrolment. However, this more in-depth qualitative aspect is not included in this trial due to resource constraints. Including a qualitative component would have also enabled further exploration of the trial flow and processes from the experiences of those who took part, but for reasons outlined, this was not a facet we could include and a limitation of this protocol.

This study will be invaluable to confirm whether our planned approach is suitable and will generate data that will facilitate the final size and design of a definitive UK multi-centre RCT.

## Additional file

Additional file 1: Example of Consent Form. (DOC 123 kb)

## Abbreviations

AE: Adverse event
AIR: Ankle injury rehabilitation
CRF: Clinical reporting form
ISRCTN: International standard randomised controlled trial number
MOXFQ: Manchester-Oxford Foot and Ankle Questionnaire
NHS: National Health Service
NIHR: National Institute for Health Research
NRES: National Research Ethics System
OMAS: Olerud and Molander Ankle Score
ORIF: Open reduction and internal fixation
REC: Research Ethics Committee
RCT: Randomised Controlled Trial
SAE: Serious Adverse Event

## References

[1] Hospital episode statistics 2013-14: http://www.hscic.gov.uk/catalogue/PUB16719

[2] Donken CCMA, Al-Khateeb H, Verhofstad MHJ, van Laarhoven CJHM. Surgical versus conservative treatment for ankle fractures in adults. Cochrane Database Syst Rev, 2012. 10.1002/14651858.CD008470.pub2 PMC12145951 22895975

[3] McPhail SM, Dunstan J, Canning J, Haines T (2012). Life impact of ankle fractures: qualitative analysis of patient and clinician experiences. BMC Musculoskelet Disord.

[4] Hedstrom M (1994). Ahl, and Dalen N. Early postoperative ankle exercise. A study of postoperative lateral malleolar fractures. Clin Orthop Relat Res.

[5] Lin CW, Donkers NAJ, Refshauge KM, Beckenkamp PR, Khera K, Moseley AM. Rehabilitation for ankle fractures in adults. Cochrane Database Syst Rev, 2012. Issue 11. Art. No.: CD005595. doi:10.1002/14651858.CD005595.pub3. 10.1002/14651858.CD005595.pub3 23152232

[6] Willett KM, Gray B, Moran CG, Giannoudis P, Pallister I (2010). Orthopaedic trauma research priority-setting exercise and development of a research network. Injury.

[7] Morley D, Jenkinson C, Doll H, Lavis G, Sharp R, Cooke P, Dawson J (2013). The Manchester-Oxford Foot Questionnaire (MOXFQ): development and validation of a summary index score. Bone Joint Res.

[8] Rabin R, de Charro F (2001). EQ-5D: a measure of health status from the EuroQol Group. Ann Med.

[9] Mont MA, Sedlin ED, Weiner LS, Miller AR (1992). Postoperative radiographs as predictors of clinical outcome in unstable ankle fractures. J Orthop Trauma.

[10] Mapstone J, Elbourne D and Roberts I. Strategies to improve recruitment to research studies. Cochrane Database Syst Rev. 2010. Issue 4. Art. No.: MR000013. doi:10.1002/14651858.MR000013.pub5. 10.1002/14651858.MR000013.pub3 17443634

[11] Sim J, Lewis M (2012). The size of a pilot study for a clinical trial should be calculated in relation to considerations of precision and efficiency. J Clin Epidemiol.

[12] Julious SA (2005). Sample size of 12 per group rule of thumb for a pilot study. Pharm Stat.

[13] Baraza N, Lever, Waight and Dhukaram V. The home therapy ankle pathway: a novel and cost effective method of initial management of ankle fractures. Bone Joint Surg, 2013. 95: Supp 21.

[14] Cooke MW, Marsh JL, Clark M, Nakash R, Jarvis RM, Hutton JL, Szcxepura A, Wilson S, Lamb SE. Treatment of severe ankle sprain: a pragmatic randomised controlled trial comparing the clinical effectiveness and cost effectiveness of three types of mechanical ankle support with tubular bandage. The CAST trial. Health Technol Assess. 2009;13(13):iii, ix–x, 1–121. 10.3310/hta13130 19232157

[15] Berntsen GK, Fonnebo V, Tollan A, Sogaard A, Magnus J (2001). Forearm bone mineral density by age in 7,620 men and women: the Tromso study, a population-based study. Am J Epidemiol.

[16] Court-Brown CM, Rimmer S, Prakash U, McQueen MM (1998). The epidemiology of open long bone fractures. Injury.

