# Supplementary material for: A protocol for a feasibility randomised controlled trial to assess the difference between functional bracing and plaster cast for the treatment of ankle fractures
Source: Pilot Feasibility Stud. 2017 Mar 1;3:11. doi: 10.1186/s40814-017-0125-z (PMC5331699; doi:10.1186/s40814-017-0125-z)
Supplement: Additional file 1: — Example of Consent Form. (DOC 123 kb) [file 40814_2017_125_MOESM1_ESM.doc]

**Additional file 1: Example of Consent Form**
